# Alpha-Ketoglutarate Attenuates UVB-Induced Skin Photoaging by Restoring Mitochondrial Redox Homeostasis

**DOI:** 10.3390/antiox15070845

**Published:** 2026-07-04

**Authors:** Wenrui Zhang, Yijia Zhang, Xinyuan Wang, Yujuan Chen, Yixuan Li, Yanan Sun

**Affiliations:** 1 Department of Nutrition and Health, China Agricultural University, Beijing 100193, China; wrzhang2021@163.com (W.Z.);; 2 College of Food Science and Nutritional Engineering, China Agricultural University, Beijing 100193, China

**Keywords:** α-ketoglutarate, skin photoaging, mtROS, AMPK, HIF-1α, α-KGDD

## Abstract

Chronic ultraviolet B (UVB) radiation drives cutaneous photoaging—clinically manifesting as erythema, edema, scaling, deep wrinkling, loss of elasticity, and barrier disruption—through mitochondrial reactive oxygen species (mtROS) overproduction and quality-control failure. Here we identify α-ketoglutarate (AKG; also known as 2-oxoglutarate), a TCA-cycle intermediate and essential co-substrate for α-ketoglutarate-dependent dioxygenases (α-KGDDs), as a metabolic corrector of mitochondrial redox homeostasis in UVB-induced photoaging. In a 10-week chronic UVB SKH1 hairless mouse model, microneedle-assisted transdermal delivery of AKG dose-dependently attenuated macroscopic erythema, scaling, and erosive lesions, restored skin barrier function and dermal elasticity, preserved epidermal–dermal architecture, and protected collagen and elastic fiber integrity, with efficacy comparable to all-trans retinoic acid. Mechanistically, AKG reactivated α-KGDD/prolyl hydroxylase (PHD) catalytic function and promoted proteasomal clearance of aberrantly stabilized HIF-1α under normoxia; this was accompanied by restored AMPK Thr172 phosphorylation downstream of constitutive LKB1 and recovery of PGC-1α-driven mitochondrial biogenesis. AKG preferentially attenuated mitochondrial superoxide over total cellular ROS through a co-substrate-mediated mechanism distinct from direct radical scavenging, and its protective effects were largely abrogated by DMOG (an α-KGDD inhibitor) or compound C (an AMPK inhibitor). These findings position AKG, delivered via microneedle-assisted topical application, as a candidate metabolite-based intervention targeting the α-KGDD/HIF-1α/AMPK axis for photoaging.

## 1. Introduction

Cutaneous photoaging, driven predominantly by chronic ultraviolet B (UVB) radiation, is one of the most prevalent dermatological conditions worldwide and a major risk factor for skin malignancies [1,2]. Clinically, UVB-damaged skin manifests as erythema, edema, deep wrinkles, loss of elasticity, and impaired barrier function, collectively imposing substantial dermatological and psychosocial burdens [3,4]. Although topical retinoids such as all-trans retinoic acid (ATRA) remain the clinical gold standard for photoaging intervention, their utility is constrained by frequent cutaneous irritation, photosensitization, and limited applicability in sensitive populations—including individuals with reactive or compromised skin barriers, those requiring long-term maintenance therapy, and women during pregnancy or lactation [5,6]. Safer and better-tolerated alternatives for photoaging intervention thus remain a pressing dermatological priority [7].

At the subcellular level, mitochondrial dysfunction has emerged as the central pathogenic driver of UVB-induced cutaneous photoaging [8,9]. UVB photons directly damage mitochondrial DNA, disrupt electron transport chain integrity, and trigger excessive mitochondrial reactive oxygen species (mtROS) generation [10,11]. Unlike transient cytosolic oxidative bursts, UVB-induced mtROS establishes a self-amplifying loop in which damaged mitochondria produce more ROS, which in turn further damages mtDNA and electron transport chain components, ultimately driving keratinocyte senescence, dermal fibroblast dysfunction, matrix metalloproteinase activation, and collagen degradation [12,13]. Conventional water-soluble antioxidants such as N-acetylcysteine (NAC) cannot efficiently penetrate the inner mitochondrial membrane, while engineered mitochondria-targeted antioxidants such as MitoTEMPO and MitoQ act as symptomatic radical scavengers without addressing the underlying mitochondrial quality defects [14,15]. These limitations highlight an urgent need for therapeutic strategies that target the upstream causes of mitochondrial oxidative stress, with restoration of the AMP-activated protein kinase (AMPK)–peroxisome proliferator-activated receptor gamma coactivator 1-alpha (PGC-1α) mitochondrial biogenesis program—the master regulator of mitochondrial quality control in skin cells—representing a particularly attractive avenue [16,17,18].

AKG, a tricarboxylic acid (TCA) cycle intermediate, occupies a unique metabolic-signaling nexus by serving as an essential co-substrate for the α-ketoglutarate-dependent dioxygenase (α-KGDD) superfamily, which couples AKG decarboxylation to substrate hydroxylation in an Fe(II)- and O_2_-dependent manner, which includes prolyl hydroxylases (PHDs), Jumonji-domain histone demethylases, and ten-eleven translocation DNA demethylases [19,20,21]. Among α-KGDD substrates, hypoxia-inducible factor 1α (HIF-1α) is the most extensively characterized: under sufficient AKG, PHDs hydroxylate HIF-1α at conserved proline residues, recruiting the von Hippel–Lindau E3 ubiquitin ligase for proteasomal degradation [22,23]. When α-KGDD activity is compromised by oxidative stress, mitochondrial dysfunction, or AKG depletion, HIF-1α aberrantly accumulates under normoxia—a state termed pseudohypoxia—which has been increasingly recognized as a maladaptive driver of metabolic reprogramming and mitochondrial dysfunction [24,25]. Notably, exogenous AKG supplementation has been shown to extend lifespan, delay age-associated decline, and exert cytoprotective effects across model organisms and aging mice [26,27,28], yet its specific role in UVB-induced skin photoaging remains poorly characterized. Emerging evidence further suggests that aberrant HIF-1α stabilization can suppress AMPK phosphorylation and disable PGC-1α-driven mitochondrial biogenesis [29,30], raising the provocative possibility that α-KGDD inactivation and consequent HIF-1α accumulation may serve as the upstream molecular event linking UVB stress to mitochondrial biogenesis failure in keratinocytes [31,32].

We hypothesized that AKG attenuates UVB-induced skin photoaging by restoring α-KGDD/PHD catalytic activity, thereby promoting HIF-1α degradation and relieving the associated suppression of AMPK–PGC-1α/TFAM signaling to re-establish mitochondrial redox homeostasis. To test this, we first characterized the in vivo photoprotective phenotype in SKH1 mice—skin physiological indices, histology (HE, Masson), and collagen/elastic-fibre integrity—across CK, UVB, ATRA, and low/medium/high topical AKG. We then applied skin transcriptomics (RNA-seq with gene-set enrichment) to identify the pathways underlying this protection, which converged on mitochondrial oxidative-stress and HIF/AMPK-related signatures. Guided by these findings, we used a HaCaT keratinocyte model to dissect the mechanism, measuring total and mitochondrial ROS, mitochondrial membrane potential (ΔΨm) and ATP, followed by the proposed signaling nodes—α-KGDD/PHD activity, HIF-1α abundance, AMPK Thr172 phosphorylation, and PGC-1α/TFAM expression—using the pharmacological probes DMOG (α-KGDD inhibition), compound C (AMPK inhibition), and MitoTEMPO (mitochondrial antioxidant) to establish the necessity and order of each node. This sequence—in vivo phenotype → transcriptomic pathway identification → in vitro mechanistic dissection—structures the present study.

## 2. Materials and Methods

### 2.1. Reagents and Antibodies

α-ketoglutarate (AKG, #75890), dimethyloxalylglycine (DMOG, a competitive inhibitor of α-ketoglutarate-dependent dioxygenases, #D3695), compound C (CC, also known as dorsomorphin, a reversible AMPK inhibitor, #P5499), all-trans retinoic acid (ATRA, #R2625), MitoTEMPO (a mitochondria-targeted superoxide dismutase mimetic, #SML0737), and N-acetyl-L-cysteine (NAC, #A7250) were purchased from Sigma-Aldrich (St. Louis, MO, USA). Propylene glycol (PG, ≥99.5% purity, #P108208) and polyethylene glycol 400 (PEG 400, # P774684) used for the topical vehicle formulation were purchased from Aladdin Biochemical Technology Co., Ltd. (Shanghai, China).

Primary antibodies for Western blotting were obtained from Proteintech Group (Wuhan, China): anti-HIF-1α (#20960-1-AP, 1:10,000), anti-LKB1 (#10746-1-AP, 1:5000), anti-AMPKα (#10929-2-AP, 1:10,000), anti-phospho-AMPKα (Thr172) (#80209-6-RR, 1:5000), anti-ACC (#21923-1-AP, 1:10,000), anti-phospho-ACC (Ser79) (#80281-2-RR, 1:5000), anti-PGC-1α (#66369-1-Ig, 1:10,000), anti-TFAM (#82745-5-RR, 1:10,000), and anti-GAPDH (#60004-1-Ig, 1:50,000). HRP-conjugated goat anti-rabbit IgG (#SA00001-2) and goat anti-mouse IgG (#SA00001-1) (Proteintech) were used as secondary antibodies (1:10,000).

The following assay kits were employed: Reactive Oxygen Species Assay Kit (DCFH-DA, #KGA7308-100, KeyGEN BioTECH, Nanjing, China); Mitochondrial Superoxide Assay Kit (MitoSOX™ Red, #S0061S), Enhanced Mitochondrial Membrane Potential Assay Kit (JC-1, #C2003S), Enhanced ATP Assay Kit (#S0027), BCA Protein Assay Kit (#P0012), and Hoechst 33342 (#C1022) (all from Beyotime Biotechnology, Shanghai, China); Superoxide Dismutase (SOD, #A001-3-2), Catalase (CAT, #A007-1-1), and Malondialdehyde (MDA, #A003-1-2) assay kits (Nanjing Jiancheng Bioengineering Institute, Nanjing, China). All other chemicals and solvents were of analytical grade. For microneedle-assisted transdermal delivery in vivo, sterile titanium-alloy microneedle dermal rollers (Hauros brand, 540 needles per drum, 0.2 mm needle length; Hebei Xiongan Qisen Medical Equipment Co., Ltd., Xiongan New Area, Hebei, China) were used.

### 2.2. Animals and Ethics Statement

Female SKH1 hairless mice (6–8 weeks old, 18–20 g) were purchased from Charles River Laboratories Co., Ltd. (Beijing, China). All animal procedures were conducted in accordance with the National Institutes of Health Guide for the Care and Use of Laboratory Animals (8th edition, 2011) and reported in compliance with the ARRIVE 2.0 guidelines. The protocol was approved by the Laboratory Animal Welfare and Animal Experimental Ethics Committee of Beijing Gushi Test Technology Co., Ltd. (the animal experimentation facility affiliated with the Department of Nutrition and Health, China Agricultural University, Beijing, China), under Approval No. GSCS-2025-011 (approved on 26 June 2025). Mice were individually housed in ventilated cages under specific pathogen-free conditions with a 12-h light/dark cycle, 22 ± 2 °C ambient temperature, and 50 ± 10% relative humidity. Standard rodent chow and autoclaved water were provided ad libitum. Mice were acclimatized for one week before the experiments. Sample size (*n* = 6 per group) was determined based on previous photoaging studies [33] and a power analysis assuming 30% mean difference and 20% standard deviation between groups (α = 0.05, power = 0.8). Predefined humane endpoints (≥20% body-weight loss, severe systemic illness, deep ulceration >1 cm^2^ or secondary infection, or inability to access food/water) were monitored daily; no animal reached these criteria during the study.

### 2.3. UVB Irradiation Protocol (In Vivo)

A bank of three UVB fluorescent lamps (TL 40W/12 RS SLV/25, Philips, Amsterdam, The Netherlands; peak emission at 312 nm) was positioned 30 cm above the dorsal surface of mice to provide uniform irradiation. UVB intensity was calibrated before each session using a UV radiometer (UV-B probe, Photoelectric Instrument Factory of Beijing Normal University, Beijing, China) at a constant irradiance of 0.225 mW/cm^2^. The escalating dosimetry schedule, adapted from established murine photoaging protocols [34], was initiated at 1 minimal erythemal dose (MED; 1 MED = 100 mJ/cm^2^) and increased by 1 MED per week to a maintenance dose of 4 MED (400 mJ/cm^2^), which was sustained for the remainder of the study. Irradiation was administered three times per week (Monday, Wednesday, Friday) for 10 consecutive weeks. Non-irradiated control mice were handled identically without UVB exposure to control for handling stress.

### 2.4. Topical Formulation, Microneedle-Assisted Transdermal Delivery, and Treatment Groups (In Vivo)

After acclimatization, mice were randomly assigned (using a random-number generator) into six groups (*n* = 6 per group): control (CK; blank vehicle + sham irradiation); UVB model (M; blank vehicle + UVB); positive control (ATRA; 0.05% *w*/*w* all-trans retinoic acid + UVB) [34]; low-dose AKG (LAKG; 1% *w*/*w* AKG + UVB); medium-dose AKG (MAKG; 2% *w*/*w* AKG + UVB); and high-dose AKG (HAKG; 3% *w*/*w* AKG + UVB).

The topical vehicle consisted of 40% (*w*/*w*) propylene glycol, 30% (*w*/*w*) polyethylene glycol 400, and 30% (*w*/*w*) deionized water, adapted from previously validated topical formulations for hairless mouse photoaging studies [35,36,37]. AKG was first dissolved in the deionized water phase at the corresponding concentration (1%, 2%, or 3% *w*/*w*), and the pH was adjusted to 7.2–7.4 using 1 M NaOH to neutralize the dicarboxylate groups of AKG. ATRA formulations were prepared by first dissolving ATRA in propylene glycol (protected from light) before sequential addition of polyethylene glycol 400 and the aqueous phase. All formulations were freshly prepared weekly and stored at 4 °C, protected from light in amber glass containers.

Microneedle-Assisted Transdermal Delivery Protocol. To enhance cutaneous penetration of the hydrophilic AKG molecule and to standardize delivery conditions across groups, a microneedle-assisted transdermal delivery protocol was applied uniformly to all six experimental groups, with the only between-group difference being the composition of the topically applied formulation. This design ensured that any procedure-related effect of microneedle rolling on epidermal regeneration, collagen deposition, or inflammatory signaling was equally distributed across all groups and therefore controlled for in between-group comparisons. Approximately 30 min before each UVB irradiation session, mice were gently restrained without anesthesia, and 100 μL of the appropriate formulation was uniformly applied to the dorsal skin (~6 cm^2^) using a calibrated micropipette. Immediately following formulation application, a sterile titanium-alloy microneedle dermal roller (Hauros, specifications in Section 2.1) was applied with light, uniform pressure and rolled 10 times in each of four directions (40 passes total) to generate transient epidermal microchannels; adequate microchannel formation was confirmed by fine, evenly distributed pinpoint erythema without macroscopic bleeding. Before use, each roller was sterilized in 75% ethanol for 30 min and air-dried in a laminar flow hood; a new, unused sterile roller was used for each animal at each session, and used rollers were discarded after each session. The procedure was performed three times per week (Monday, Wednesday, Friday) throughout the 10-week study period.

Body weight was recorded weekly. Macroscopic dorsal skin photographs were captured weekly under standardized lighting using a digital camera (Canon EOS series). Skin appearance was inspected daily; transient pinpoint erythema typically resolved within hours of microneedle application, and no sustained adverse events were observed in any group. All investigators performing measurements and scoring were blinded to the group assignments.

### 2.5. Skin Biophysical Measurements

At the end of the 10-week experimental period and 24 h after the final UVB exposure, non-invasive skin biophysical parameters were measured on the dorsal skin of anesthetized mice (isoflurane inhalation) using a multi-probe adapter system (MPA, Courage + Khazaka Electronic GmbH, Cologne, Germany) in a temperature- and humidity-controlled room. Parameters assessed included transepidermal water loss (TEWL, Tewameter^®^ TM 300), stratum corneum hydration (Corneometer^®^ CM 825), sebum content (Sebumeter^®^ SM 815), skin elasticity (Cutometer^®^ MPA 580), skin anisotropy (Reviscometer^®^ RVM 600), and erythema index (Mexameter^®^ MX 18), all of which were obtained from Courage + Khazaka Electronic GmbH, Cologne, Germany. Each parameter was measured at three randomized sites per mouse and averaged by a single blinded operator.

### 2.6. Macroscopic Skin Scoring

Photoaging severity was evaluated weekly using a semi-quantitative 0–6 scoring system [38,39] by two independent blinded observers, assessing erythema severity (0–2), scaling/dryness (0–2), and wrinkling (0–2). Inter-observer agreement (intraclass correlation coefficient > 0.85) was confirmed before analysis.

### 2.7. Histological Analysis

Following euthanasia by exsanguination under deep isoflurane anesthesia, dorsal skin samples were fixed in 4% paraformaldehyde, processed through graded ethanol and xylene, paraffin-embedded, and sectioned at 5 μm using a rotary microtome (Leica RM2235). Sections were stained with hematoxylin and eosin (Servicebio, #G1004 and #G1001) for epidermal/dermal thickness measurement, Masson’s trichrome (Servicebio, #G1006) for collagen volume fraction quantification, and Verhoeff–Van Gieson (Servicebio, #G1042) for elastic fiber density assessment, all performed according to the manufacturers’ protocols. Quantitative morphometric analyses were performed in five randomly selected high-power fields (×400) per section using ImageJ software (version 1.54j; NIH, Bethesda, MD, USA) by observers blinded to group assignments.

### 2.8. Immunohistochemical (IHC) Staining

Deparaffinized sections underwent heat-induced antigen retrieval in 10 mM sodium citrate buffer (pH 6.0), endogenous peroxidase quenching with 3% H_2_O_2_, and blocking with 5% normal goat serum (Beyotime, #C0265). Sections were incubated overnight at 4 °C with primary antibodies against Ki67 (Proteintech, #27309-1-AP, 1:500) or p63 (Proteintech, #12143-1-AP, 1:200), followed by biotinylated secondary antibody and streptavidin-HRP (SP-9001, ZSGB-BIO), and visualized with DAB substrate (ZSGB-BIO, #ZLI-9018). Negative controls were processed with primary antibody omission. Ki67-positive basal cells and p63-positive epidermal cells were quantified in five high-power fields (×400) per section by two blinded observers.

### 2.9. RNA Sequencing (RNA-seq) and Bioinformatic Analysis

Total RNA was extracted from dorsal skin tissues (CK, UVB, and HAKG groups; *n* = 3 per group) using TRIzol reagent (Invitrogen, #15596018), and only samples with RNA Integrity Number (RIN) ≥ 7.0 (Agilent 2100 Bioanalyzer; Agilent Technologies, Santa Clara, CA, USA) were used for library construction with the NEBNext^®^ Ultra™ RNA Library Prep Kit for Illumina^®^ (New England Biolabs, Ipswich, MA, USA, #E7530). Paired-end sequencing (150 bp) was performed on an Illumina NovaSeq 6000 platform (Novogene, Beijing, China). Clean reads (after FastQC v0.11.9 quality control and Trimmomatic v0.39 trimming) were aligned to the mouse reference genome (GRCm39/mm39) using HISAT2 (v2.2.1), and gene-level counts were obtained with featureCounts (Subread v2.0.3) using Ensembl annotation (release 108). Differentially expressed genes (DEGs) were identified using DESeq2 (v1.34.0) with the criteria |log_2_FC| > 1 and FDR < 0.05 (Benjamini–Hochberg correction). GO and KEGG enrichment analyses were performed using clusterProfiler (v4.2.0); GSEA was conducted using GSEA software (v4.2.3) against MSigDB Hallmark and KEGG collections, with NES > 1.5 and FDR < 0.05 considered significant. RNA-seq data have been deposited in the National Genomic Data Center (accession PRJCA063446).

### 2.10. Oxidative Stress Biomarkers in Skin Tissue

Frozen dorsal skin tissues were homogenized in ice-cold PBS (10% *w*/*v*), and supernatants (12,000× *g*, 15 min, 4 °C) were assayed for superoxide dismutase (SOD, #A001-3-2; xanthine oxidase/hydroxylamine method), catalase (CAT, #A007-1-1; ammonium molybdate method), and malondialdehyde (MDA, #A003-1-2; thiobarbituric acid method) using commercial kits (Nanjing Jiancheng Bioengineering Institute) per manufacturers’ instructions. Activities were normalized to total protein concentration (BCA assay).

### 2.11. Cell Culture and UVB Irradiation

The human immortalized keratinocyte cell line HaCaT was obtained from the Cell Bank of the Chinese Academy of Sciences (Shanghai, China), authenticated by short tandem repeat (STR) profiling, and verified mycoplasma-negative. Cells were cultured in DMEM (Gibco, Thermo Fisher Scientific, Waltham, MA, USA) supplemented with 10% fetal bovine serum(Gibco; Thermo Fisher Scientific) and 1% penicillin–streptomycin(Gibco; Thermo Fisher Scientific) at 37 °C in 5% CO_2_, and used between passages 3 and 15.

For UVB irradiation, the culture medium was replaced with a thin layer of PBS, and cells were exposed to a single dose of 30 mJ/cm^2^ UVB (TL 20W/12 RS SLV lamp, Philips; peak emission 312 nm), selected based on dose–response experiments yielding approximately 70% viability (Supplementary Figure S1A) [10,40]. Immediately after irradiation, PBS was replaced with fresh complete medium containing the indicated treatments. Sham-irradiated controls underwent identical medium replacement without UVB exposure.

### 2.12. Drug Treatments (In Vitro)

AKG was dissolved in complete culture medium (pH 7.2–7.4) and used at 4 mM, the optimal protective concentration determined by parallel CCK-8 dose–response and rescue experiments (Supplementary Figure S1B,C). DMOG (1 mM, dissolved in DMSO) [25] and compound C (10 μM, dissolved in DMSO) were used as competitive α-KGDD and AMPK inhibitors, respectively. MitoTEMPO (10 μM) and N-acetyl-L-cysteine (NAC, 5 mM) were used as mitochondria-targeted and non-selective antioxidant comparators [15]. The final DMSO concentration in all treatment groups did not exceed 0.1% (*v*/*v*), with equivalent DMSO added to all controls as vehicle. Cytotoxicity controls confirming the absence of independent toxicity at the working concentrations of DMOG and compound C are shown in Supplementary Figure S1D.

Pharmacological inhibitors (DMOG or compound C) were pre-incubated with cells for 1 h prior to UVB irradiation. AKG and antioxidant comparators were administered immediately after UVB exposure in fresh medium. Cells were harvested at 24 h post-irradiation for protein extraction or fluorescence imaging, unless otherwise indicated. Detailed experimental groupings for each functional assay are described in the corresponding Results sections.

### 2.13. Cell Viability Assay (CCK-8)

HaCaT cells (8 × 10^3^ cells/well in 96-well plates) were treated as indicated and assessed for viability using Cell Counting Kit-8 (Dojindo Laboratories, Kumamoto, Japan) per the manufacturer’s instructions, with absorbance read at 450 nm (SpectraMax i3x, Molecular Devices, San Jose, CA, USA). Each condition was performed in six replicate wells across three independent experiments.

### 2.14. Mitochondrial and Redox Imaging Assays

Total intracellular ROS (DCFH-DA, 10 μM, 30 min), mitochondrial superoxide (MitoSOX™ Red, 5 μM, 10 min), and mitochondrial membrane potential (JC-1, Beyotime #C2003S, 10 μg/mL, 20 min) were detected in HaCaT cells per the manufacturer’s protocols. Nuclei were counterstained with Hoechst 33342 (Beyotime #C1022, 10 μg/mL). Fluorescence images were captured with an inverted fluorescence microscope (Olympus IX73; excitation/emission: 488/525 nm for DCFH-DA, 510/580 nm for MitoSOX, 514/529 nm for JC-1 monomer, 585/590 nm for JC-1 aggregates, 350/461 nm for Hoechst). Mean fluorescence intensity, normalized to nuclei count, was quantified across at least five random fields per condition using ImageJ. The selectivity index (SI) for compartmental ROS suppression was calculated as: SI = (% MitoSOX reduction vs. UVB)/(% DCFH-DA reduction vs. UVB), with statistical comparison against the non-selective baseline (SI = 1.0) by one-sample *t*-test.

Intracellular ATP was measured using the Enhanced ATP Assay Kit (Beyotime #S0027) based on a firefly luciferase–luciferin bioluminescence reaction. HaCaT cells (3 × 10^5^/well in 6-well plates) were lysed in ATP lysis buffer; supernatants were combined with detection reagent and luminescence was measured on a SpectraMax i3x microplate reader. ATP concentrations were calculated from a standard curve (0.01–10 μM), normalized to total protein (BCA), and expressed as a percentage of CK.

### 2.15. Western Blotting

Cell or skin tissue lysates were prepared in RIPA buffer supplemented with protease and phosphatase inhibitor cocktails. Equal amounts of protein (20–40 μg) determined by BCA assay were resolved by 8–12% SDS-PAGE, transferred to 0.22 or 0.45 μm PVDF membranes, and blocked with 5% non-fat milk (or 5% BSA for phospho-specific antibodies) in TBST. Membranes were incubated overnight at 4 °C with the primary antibodies listed in Section 2.1, followed by the corresponding HRP-conjugated secondary antibodies (Proteintech, 1:10,000). Bands were visualized with ECL substrate (Millipore #WBKLS0500) on a Tanon 5200 imaging system and quantified by ImageJ; phosphorylated proteins were normalized to their corresponding total protein, and total proteins to GAPDH. All experiments were performed with at least three biologically independent replicates.

### 2.16. Statistical Analysis

Quantitative data are presented as mean ± SEM (in vivo experiments) or mean ± SD (in vitro experiments), with biological replicate numbers (*n*) indicated in figure legends. Statistical analyses were performed using GraphPad Prism v9.5.1 (GraphPad Software, San Diego, CA, USA). Normality and homogeneity of variance were assessed by Shapiro–Wilk and Levene’s tests, respectively. Multi-group comparisons used one-way ANOVA followed by Tukey’s HSD post hoc test (or Welch’s ANOVA with Dunnett’s T3 for unequal variances). Paired *t*-tests were applied for within-group compartmental comparisons (cytosolic vs. mitochondrial ROS), and one-sample *t*-tests for selectivity index comparisons against the non-selective baseline (SI = 1.0). A two-tailed *p* < 0.05 was considered significant. Significance is denoted as: * *p* < 0.05, ** *p* < 0.01, *** *p* < 0.001 vs. CK; # *p* < 0.05, ## *p* < 0.01, ### *p* < 0.001 vs. UVB; + *p* < 0.05, ++ *p* < 0.01, +++ *p* < 0.001 between specified groups. All microscopy-based quantification was performed by observers blinded to group assignments.

## 3. Results

### 3.1. Topical AKG Administration Ameliorates UVB-Induced Skin Macro-Damage and Physiological Dysfunction

**Figure 1:**
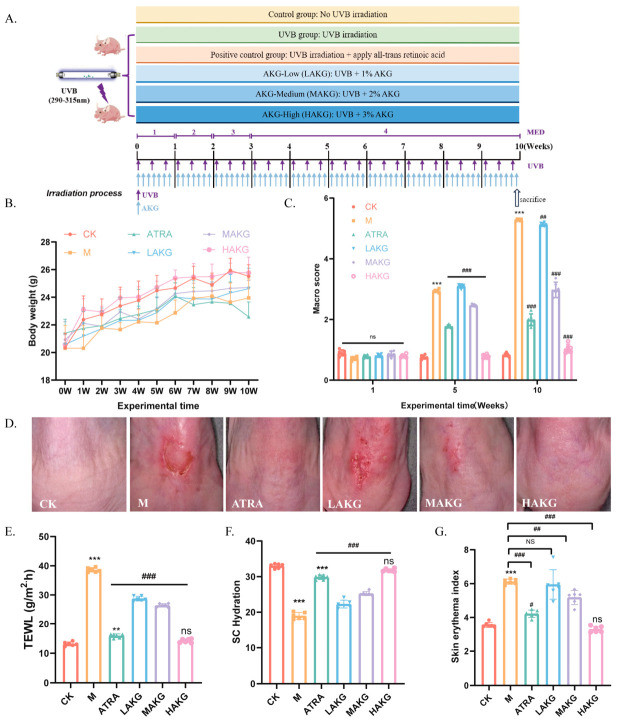
Topical AKG administration mitigates macroscopic skin damage and physiological dysfunction in UVB-irradiated hairless mice. (**A**) Schematic workflow of the 10-week chronic UVB irradiation protocol and topical treatment regimens across the six animal groups (Control/CK, Model/M, ATRA positive control, LAKG, MAKG, and HAKG). (**B**) In vivo body weight monitoring over the 10-week experimental timecourse. (**C**) Macro scoring evaluation of skin damage at weeks 0, 3, 5, and 10. (**D**) Representative macroscopic photographs illustrating the dorsal skin surface of hairless mice from each designated group at the end of week 10. Quantitative analysis of vital skin physiological parameters at the 10-week endpoint: (**E**) Transepidermal water loss (TEWL), (**F**) stratum corneum (SC) hydration, and (**G**) skin erythema index. Data are presented as mean ± SEM (*n* = 6). *** *p* < 0.001 vs. CK group; # *p* < 0.05, ## *p* < 0.01, ### *p* < 0.001 vs. UVB/M group; ns, not significant.

To investigate the protective effects of AKG against photoaging, SKH1 hairless mice were subjected to a 10-week chronic UVB irradiation protocol across six distinct experimental groups, as detailed in Section 2.4 and schematically illustrated in Figure 1A. Chronic UVB exposure led to a slight, non-significant restriction in body weight gain compared to the untreated control (CK) group, which was not markedly altered by topical treatments (Figure 1B). Macroscopically, UVB-irradiated mice exhibited profound skin photoaging phenotypes, characterized by severe erythema, coarse wrinkling, skin sagging, and occasional excoriations (Figure 1D). Consequently, the skin macro score was drastically elevated in the vehicle-treated UVB group (M) compared with the CK group (Figure 1C). Conversely, topical application of AKG dose-dependently mitigated these macroscopic lesions. Notably, mice treated with high-dose AKG (HAKG) demonstrated smooth skin with noticeably shallow wrinkles, achieving a therapeutic efficacy comparable to the positive control group treated with all-trans retinoic acid (ATRA) (Figure 1C,D).

To evaluate skin barrier function and physiological status, we assessed transepidermal water loss (TEWL), stratum corneum (SC) hydration, and the skin erythema index. Chronic UVB exposure induced severe skin barrier disruption, manifested by a profound elevation in TEWL values (Figure 1E) and a significant depletion in SC hydration levels compared to the CK group (Figure 1F). Topical administration of AKG substantially reversed these physiological impairments in a dose-dependent manner. In the HAKG group, TEWL values were markedly attenuated (Figure 1E) and SC moisture content experienced a substantial recovery toward baseline levels (Figure 1F). Furthermore, the UVB-induced increase in the skin erythema index was significantly suppressed by AKG treatments (Figure 1G). Secondary physiological parameters, including sebum, anisotropy, and elastic recovery, were reallocated to the Supplementary Materials (Figure S2).

### 3.2. AKG Alleviates Histopathological Alterations and Cutaneous Oxidative Stress in Vivo

**Figure 2:**
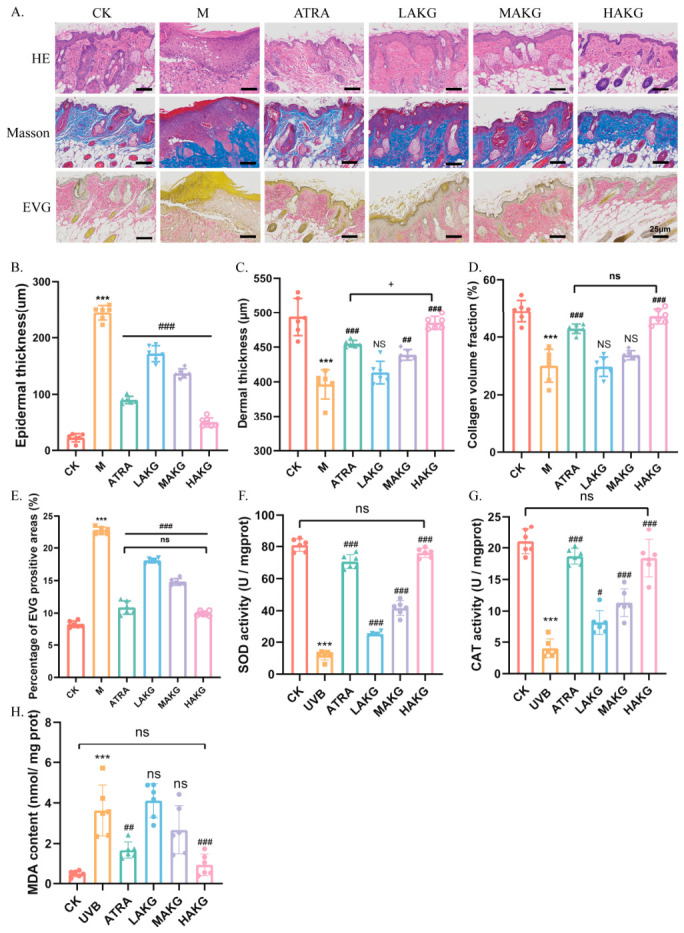
AKG alleviates histopathological alterations, matrix degradation, and cutaneous oxidative stress in vivo. (**A**) Representative microscopic images of mouse skin tissue sections subjected to Hematoxylin and Eosin (H&E, scale bar = 25 μm), Masson’s trichrome (scale bar = 25 μm), and Verhoeff–Van Gieson (EVG, scale bar = 25 μm) staining. Morphometric quantifications of structural changes: (**B**) Epidermal thickness (μm), (**C**) Dermal thickness (μm), (**D**) dermal collagen volume fraction (%), and (**E**) percentage of EVG-positive elastic fiber area (%). Evaluation of cutaneous redox status and lipid peroxidation parameters: (**F**) Superoxide dismutase (SOD) activity, (**G**) catalase (CAT) activity, and (**H**) malondialdehyde (MDA) content. Data are expressed as mean ± SD (*n* = 6). *** *p* < 0.001 vs. CK group; # *p* < 0.05, ## *p* < 0.01, ### *p* < 0.001 vs. UVB/M group;+p<0.05 for HAKG group vs. ATRA group; ns, not significant.

Histological evaluations via Hematoxylin and Eosin (H&E), Masson’s trichrome, and Verhoeff–Van Gieson (EVG) staining were performed to evaluate structural changes in the dermis and epidermis (Figure 2A). H&E staining revealed that chronic UVB irradiation induced severe epidermal hyperplasia, resulting in a dramatic increase in epidermal thickness compared to the CK group (Figure 2A,B). Topical application of AKG markedly attenuated this hyperplastic response, with the MAKG and HAKG groups displaying a significant reduction in epidermal thickness (Figure 2B). In the dermal compartment, Masson’s and EVG staining demonstrated that UVB exposure caused extensive degradation, fragmentation, and disorganized alignment of collagen and elastic fibers, culminating in a significant decrease in dermal thickness (Figure 2A,C), collagen volume fraction (Figure 2D), and the percentage of EVG-positive elastic areas (Figure 2E). AKG intervention dose-dependently countered these matrix-degrading effects, substantially preserving dermal thickness and maintaining the density and structural integrity of the extracellular matrix fibers (Figure 2A,C–E).

Given that oxidative stress is a primary driver of photoaging, we next evaluated cutaneous antioxidant enzyme activities and lipid peroxidation products. Chronic UVB exposure severely compromised the skin’s endogenous antioxidant defense, as evidenced by a profound reduction in superoxide dismutase (SOD) (Figure 2F) and catalase (CAT) activities (Figure 2G) compared to the CK group. This was accompanied by a significant accumulation of malondialdehyde (MDA), reflecting aggravated lipid peroxidation (Figure 2H). Remarkably, AKG administration effectively restored the cutaneous redox balance. Treatment with AKG resulted in a significant, dose-dependent restoration of both SOD and CAT activities (Figure 2F,G), alongside a marked reduction in MDA content (Figure 2H).

### 3.3. AKG Restores Epidermal Cell Proliferation and Modulates Photoaging-Related Metabolic Pathways

**Figure 3:**
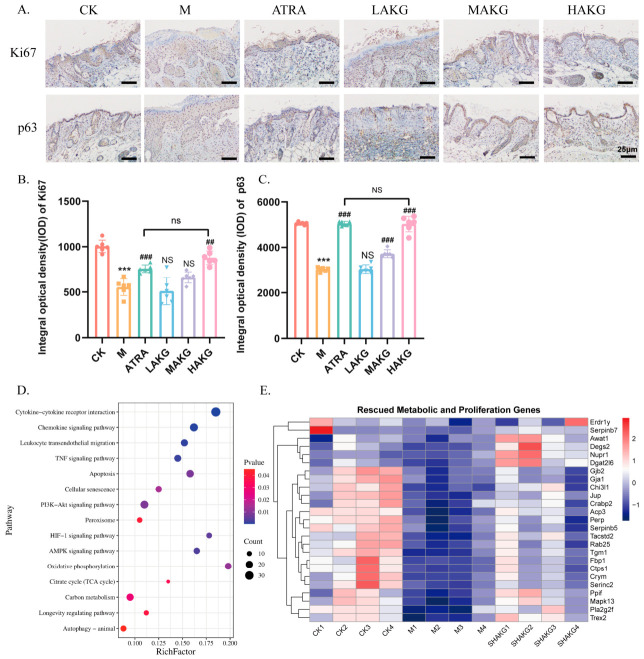
AKG restores basal cell proliferation and modulates photoaging-related transcriptional networks. (**A**) Representative immunohistochemical (IHC) staining images profiles for proliferation markers Ki67 and p63 within the basal layer of the mouse epidermis (scale bar = 25 μm). Quantitative evaluation based on the integral optical density (IOD) for (**B**) Ki67 and (**C**) p63 immunoreactivity. Transcriptomic landscape determined via skin RNA-seq: (**D**) Bubble plot detailing the significant KEGG pathway enrichment terms altered under UVB stress and rescued by AKG. (**E**) Comprehensive clustering heatmap illustrating the expression patterns of specific rescued genes mapped to metabolic, proliferative, and mitochondrial homeostasis processes. Data are presented as mean ± SD. *** *p* < 0.001 vs. CK group; ## *p* < 0.01, ### *p* < 0.001 vs. UVB/M group; ns, not significant.

To explore the cellular mechanisms underlying the observed epidermal rejuvenation, immunohistochemical staining of proliferation markers Ki67 and p63 was conducted (Figure 3A). Quantitative analysis revealed that chronic UVB irradiation significantly reduced the integral optical density (IOD) of both Ki67-positive (Figure 3B) and p63-positive cells (Figure 3C) in the basal layer of the epidermis, indicating a state of proliferation exhaustion. Topical application of AKG significantly rescued the expression of both markers, with the MAKG and HAKG groups demonstrating robust cell proliferation in the basal layer, statistically superior to the vehicle-treated UVB group (Figure 3A–C). (Independent in vitro EdU incorporation assays were moved to Supplementary Figure S3).

To comprehensively identify the molecular pathways modulated by AKG, transcriptomic RNA-sequencing (RNA-seq) analysis was performed on mouse skin tissues. The primary data filtration, including transcriptomic volcano plots and GSEA curves, was reallocated to the Supplementary Materials (Figure S4). KEGG pathway enrichment analysis of the differentially expressed genes (DEGs) rescued by AKG treatment highlighted several crucial cellular programs, including the “Longevity regulating pathway,” “HIF-1 signaling pathway,” “AMPK signaling pathway,” and “Oxidative phosphorylation” (Figure 3D). To visualize individual gene alterations within these enriched terms, a comprehensive heatmap was generated for rescued metabolic and proliferation-associated genes (Figure 3E). The transcriptional profiling clearly showed that AKG counteracted the UVB-induced downregulation of core metabolic, mitochondrial, and proliferative transcripts, returning their expression patterns back toward the baseline state observed in the CK group (Figure 3E). These bioinformatic findings suggest that the photoprotective effects of AKG are intimately linked to the modulation of mitochondrial metabolism and energy-sensing pathways.

### 3.4. AKG Efficiently Scavenges Intracellular and Mitochondrial ROS in HaCaT Keratinocytes

**Figure 4:**
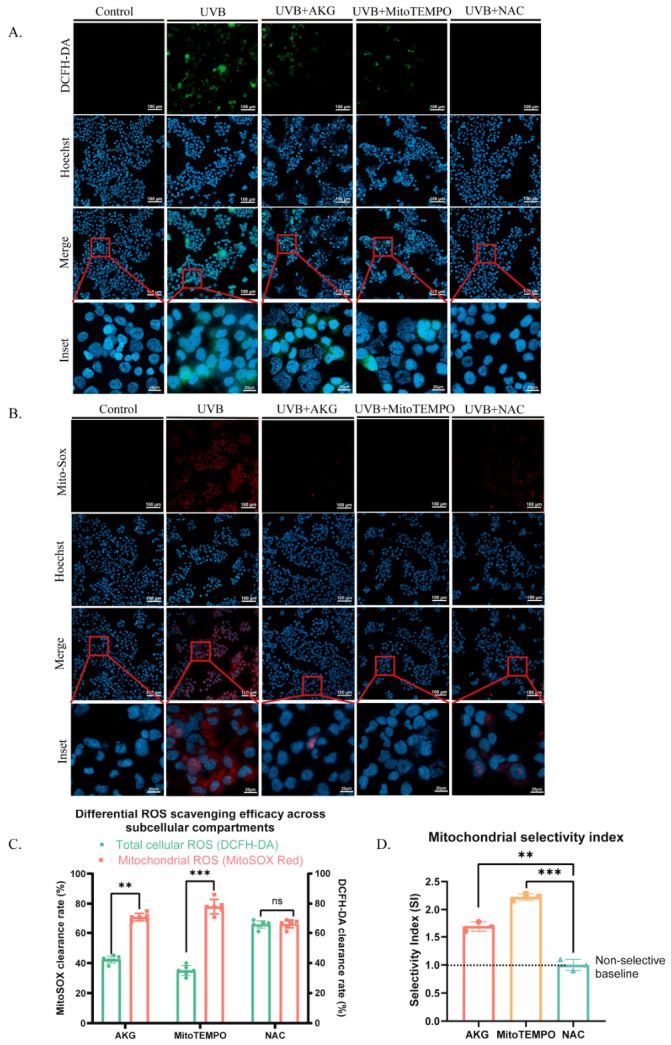
AKG efficiently and selectively scavenges intracellular and mitochondrial ROS in HaCaT cells. (**A**) Representative confocal fluorescence images of HaCaT keratinocytes stained with DCFH-DA to monitor total cellular ROS accumulation (green, scale bars: main images = 100 μm; insets = 25 μm) alongside Hoechst counterstain (blue). (**B**) Representative confocal fluorescence images of cells stained with MitoSOX Red to track mitochondrial-specific ROS levels (red, scale bars: main images = 100 μm; insets = 25 μm). Insets show magnified views of individual cellular organelle morphologies. (**C**) Quantitative analysis comparing the relative subcellular scavenging efficacy (%) across total cellular ROS and mitochondrial ROS networks among AKG, MitoTEMPO, and NAC arms. (**D**) Evaluation of the Mitochondrial Selectivity Index (SI), highlighting the preferential mitochondrial targeting capability of AKG relative to non-selective baselines. Data represent mean ± SD from three independent biological replicates. *** *p* < 0.001; ** *p* < 0.01; ns, not significant.

To further dissect the mechanisms at the cellular level, in vitro experiments were conducted utilizing human HaCaT keratinocytes. Given the vital role of oxidative species in propagating mitochondrial collapse, we evaluated the accumulation of total cellular ROS and mitochondrial-specific ROS using DCFH-DA and MitoSOX Red fluorescent probes, respectively (Figure 4A,B). UVB irradiation triggered an intense increase in both green (DCFH-DA) and red (MitoSOX) fluorescence intensities, demonstrating a massive burst of intracellular and mitochondrial oxidative stress (Figure 4A,B). Co-treatment with AKG dramatically suppressed the accumulation of both total cellular and mitochondrial ROS, with an efficacy that was comparable to, or exceeded, that of the mitochondrial-targeted antioxidant MitoTEMPO and the classic general antioxidant N-acetylcysteine (NAC) (Figure 4A,B). Quantitative assessments confirmed high ROS scavenging efficiencies for AKG across subcellular compartments, yielding a substantial clearance rate for both indicators (Figure 4C). Crucially, evaluation of the mitochondrial selectivity index (SI) revealed that AKG exhibited a highly pronounced selectivity toward scavenging mitochondrial ROS over bulk cytosolic ROS, significantly outperforming the non-selective baseline profile of NAC (Figure 4D).

### 3.5. AKG Restores Basal Mitochondrial Membrane Potential and ATP Production Without Perturbing Homeostasis

**Figure 5:**
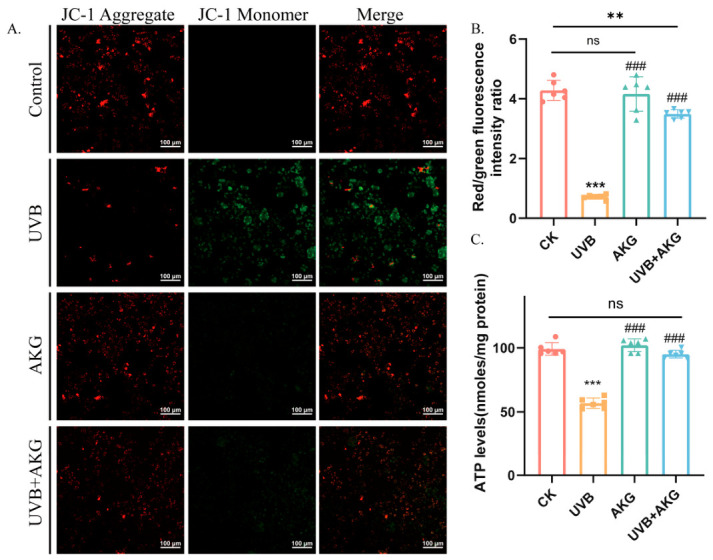
AKG maintains baseline mitochondrial polarization and ATP generation under pathological stress. (**A**) Representative ratiometric fluorescence imaging of HaCaT cells loaded with the membrane potential-sensitive dye JC-1, displaying aggregates (red, high potential), monomers (green, low potential), and merged fields under Control, UVB, AKG-alone, and UVB+AKG environments (scale bar = 100 μm). (**B**) Quantitative ratiometric analysis representing the JC-1 red/green fluorescence intensity ratio. (**C**) Measurements of intracellular ATP production levels across the designated experimental groups. Data are shown as mean ± SD (*n* = 3). ** *p* < 0.01, *** *p* < 0.001 vs. CK group; ### *p* < 0.001 vs. UVB group; ns, not significant indicating equivalence to baseline resting states.

To address the critical baseline effects of AKG and evaluate whether the molecule impacts unperturbed cells, an independent AKG-alone treatment group was established alongside the standard Control, UVB, and UVB+AKG arms. Mitochondrial membrane potential (ΔΨm) was monitored using the ratiometric fluorescent probe JC-1 (Figure 5A). In healthy control cells, JC-1 formed aggregates emitting intense red fluorescence; following UVB irradiation, the loss of (ΔΨm) shifted the dye into its monomeric form, emitting green fluorescence (Figure 5A). Consequently, the ratiometric red/green fluorescence intensity dropped dramatically in the UVB group (Figure 5B). Treatment with AKG led to a substantial recovery of the red/green ratio under UVB stress, reflecting significant protection against mitochondrial polarization loss (Figure 5B). Crucially, cells treated with AKG alone in the absence of UVB exhibited JC-1 red/green ratios that were statistically indistinguishable from the untreated CK group, confirming that AKG does not abnormally hyperpolarize or perturb basal mitochondrial status (Figure 5B).

Consistently, quantification of cellular energy currency revealed that UVB exposure led to a severe depletion of intracellular ATP levels (Figure 5C). AKG intervention significantly countered this deficit, inducing a substantial recovery of ATP production in UVB-exposed cells (Figure 5C). In parallel with the JC-1 observations, administration of AKG alone under basal conditions resulted in no significant deviation in ATP levels relative to control cells (Figure 5C).

### 3.6. AKG Regulates the α-KGDD/HIF-1α/AMPK Signaling Axis to Maintain Mitochondrial Homeostasis

**Figure 6:**
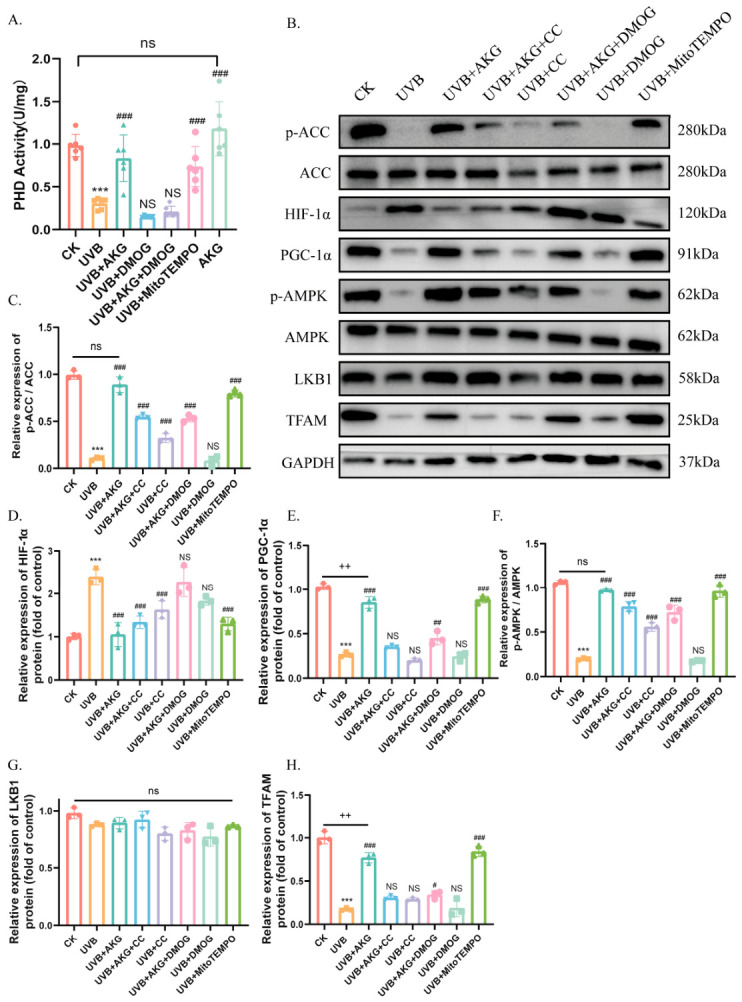
AKG regulates the α-KGDD/HIF-1α/AMPK signaling cascade under UVB irradiation. (**A**) Quantitative evaluation of prolyl hydroxylase (PHD) enzymatic activity across in vitro groups including single and combination treatments with DMOG and MitoTEMPO. (**B**) Representative Western blot bands showing the protein abundance of p-ACC, ACC, HIF-1α, PGC-1α, p-AMPK, AMPK, LKB1, and TFAM, with GAPDH serving as the internal loading control. Relative densitometric quantification charts for protein expression: (**C**) p-ACC/ACC ratio, (**D**) HIF-1α, (**E**) PGC-1α, (**F**) p-AMPK/AMPK ratio, (**G**) LKB1, (**H**) TFAM. Data represent mean ± SD (*n* = 3). *** *p* < 0.001 vs. CK group; # *p* < 0.05, ## *p* < 0.01, ### *p* < 0.001 vs. UVB group; ++ *p* < 0.01 vs. indicated comparison; ns, not significant.

To establish the exact molecular mechanism, we investigated the α-KGDD)/ PHD activity and downstream protein cascades. Quantitative enzymatic assays demonstrated that UVB irradiation or treatment with the competitive PHD inhibitor dimethyloxalylglycine (DMOG) caused a severe inhibition of PHD activity (Figure 6A). Remarkably, AKG administration significantly reactivated PHD activity under UVB stress, while AKG treatment alone maintained normal baseline levels (Figure 6A).

Western blot analyses revealed that the inhibition of PHD by UVB or DMOG resulted in a massive accumulation of hypoxia-inducible factor-1α (HIF-1α) protein (Figure 6B,D). This accumulation precisely coincided with a profound reduction in the phosphorylation of AMP-activated protein kinase (p-AMPK, Thr172) and its downstream metabolic target acetyl-CoA carboxylase (p-ACC), alongside decreased expression of core mitochondrial biogenesis drivers, including peroxisome proliferator-activated receptor gamma coactivator 1-alpha (PGC-1α) and transcription factor A, mitochondrial (TFAM) (Figure 6B,C,E,F). Notably, the abundance of the upstream kinase LKB1 remained unchanged across all groups (Figure 6B,G).

Importantly, AKG treatment or the addition of MitoTEMPO largely reversed these pathological shifts, promoting the clearance of HIF-1α, and successfully restoring AMPK/ACC phosphorylation as well as PGC-1α and TFAM expression (Figure 6B–H). To validate the regulatory link, cells were treated with the AMPK inhibitor Compound C (CC) or the PHD inhibitor DMOG alongside AKG under UVB irradiation. The introduction of CC or DMOG significantly attenuated or largely reversed the beneficial molecular effects of AKG, preventing the reactivation of AMPK signaling and suppressing downstream mitochondrial biogenesis targets (Figure 6B–H). These data present strong associative evidence that AKG coordinates mitochondrial biogenesis by regulating the PHD/HIF-1α/AMPK signaling cascade.

### 3.7. Pharmacological Interventions of the α-KGDD/HIF-1α/AMPK Axis Abrogate the Mitochondrial Protective Efficacy of AKG

**Figure 7:**
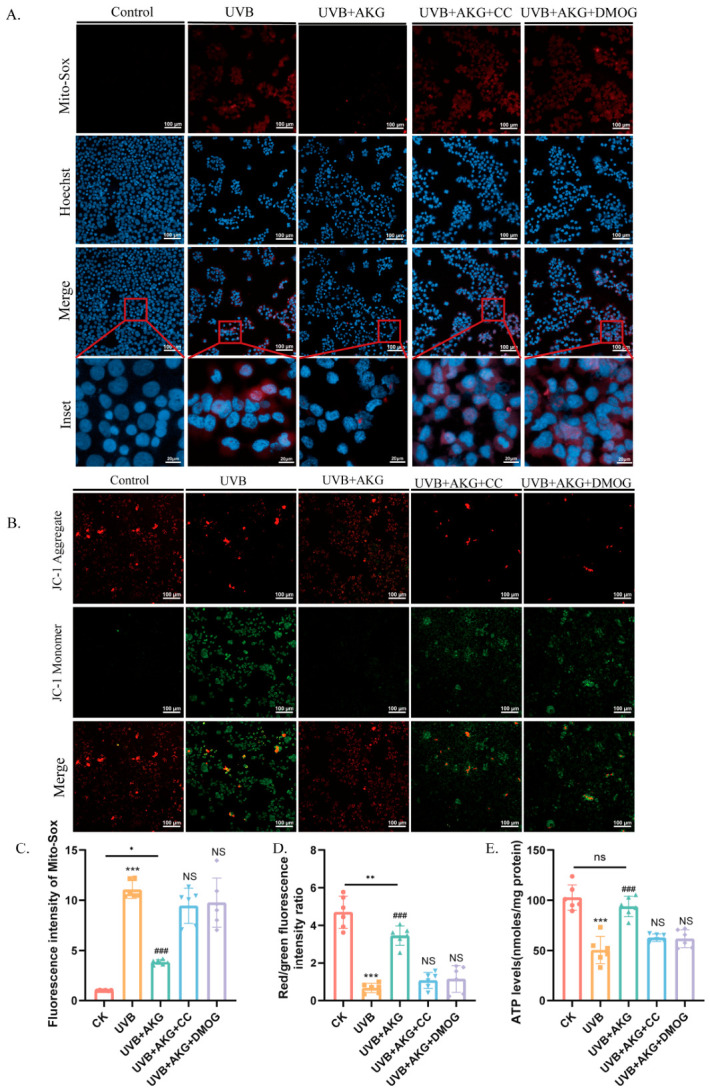
Pharmacological blockage of the regulatory axis markedly attenuates the organelle-protective efficacy of AKG. (**A**) Representative confocal micrographs of MitoSOX Red fluorescence (mitochondrial ROS, red) combined with Hoechst nuclear staining (blue) in cells co-incubated with AMPK inhibitor Compound C (CC) or PHD inhibitor DMOG (scale bars: main images = 100 μm; insets = 25 μm.). (**B**) Ratiometric JC-1 mitochondrial membrane potential imaging profiles for corresponding inhibitor co-treatments (scale bar = 100 μm). Quantitative functional metrics including: (**C**) Fluorescence intensity of MitoSOX Red, (**D**) ratiometric JC-1 red/green intensity ratio, and (**E**) intracellular ATP production levels. Data are expressed as mean ± SD (*n* = 3). * *p* < 0.05, ** *p* < 0.01, *** *p* < 0.001 vs. CK group; ### *p* < 0.001 vs. UVB group; NS, not statistically significant relative to UVB group; ns, not statistically significant relative to CK group.

To confirm whether the functional organelle protection mediated by AKG depends strictly on this signaling pathway, HaCaT cells were exposed to UVB and AKG in the presence or absence of CC and DMOG, followed by vital mitochondrial readouts. In vitro imaging via MitoSOX Red demonstrated that the robust capability of AKG to scavenge mitochondrial ROS under UVB stress was markedly attenuated by the addition of either CC or DMOG, resulting in a recurrence of high red fluorescence intensities (Figure 7A,C).

Similarly, JC-1 fluorescent imaging revealed that the AKG-mediated rescue of mitochondrial membrane potential (ΔΨm) was largely reversed upon pharmacological inhibition with CC or DMOG, as evidenced by a substantial decline in the red/green fluorescence ratio (Figure 7B,D). Furthermore, quantitative analysis of metabolic output showed that the protective effect of AKG on restoring intracellular ATP levels was significantly abrogated by both inhibitors (Figure 7E).

**Figure 8:**
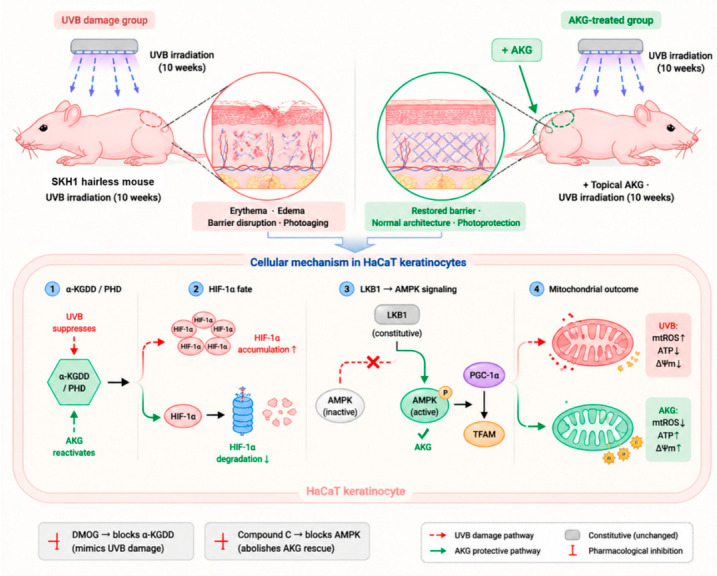
Schematic model illustrating the mechanisms of topical AKG-mediated photoprotection against UVB-induced skin photoaging. In vivo topical administration of AKG preserves skin architectural integrity, barrier parameters, and alleviates edema or erythema in hairless mice. At the cellular level within keratinocytes, UVB exposure impairs α-KGDD/PHD enzymatic activity, triggering a robust accumulation of HIF-1α. This accumulation is linked through associative mechanisms (indicated by the dashed regulatory arrow) to the suppression of AMPK Thr172 phosphorylation, subsequently dampening the downstream PGC-1α/TFAM pathway and leading to mitochondrial redox collapse (manifested by excessive mtROS, loss of ΔΨm, and ATP deficits). Exogenous supply of AKG reactivates PHD to restore HIF-1α clearance, enabling constitutive LKB1 to sustain the active AMPK network, thus maintaining mitochondrial homeostasis and photoprotection.

Together, these inhibitor experiments demonstrate thar AKG’s mitochondrial protection-encompassing superoxide suppression, membrane-potential maintenance, and ATP restoration-requires an intact α-KGDD/HIF-1α/AMPK axis. A schematic integrating the in vivo and in vitro findings into the proposed mechanism is presented in Figure 8.

## 4. Discussion

The present study identifies AKG as a metabolite-based regulator of mitochondrial redox homeostasis in UVB-induced photoaging, acting through an α-KGDD/HIF-1α–AMPK–PGC-1α/TFAM axis. Rather than scavenging radicals chemically, AKG functions as a co-substrate that restores the cell’s own mitochondrial quality-control machinery. We interpret the findings in the order in which the study progressed.

At the tissue level, microneedle-assisted topical AKG conferred dose-dependent protection against UVB photoaging in SKH1 hairless mice, preserving barrier function, epidermal–dermal architecture, and collagen/elastic-fibre organization, with high-dose AKG approaching the efficacy of the retinoid standard ATRA. AKG therefore joins the small group of metabolite-based interventions able to match retinoid-level structural protection [5,6], while its identity as an endogenous TCA-cycle metabolite suggests a potentially more favourable tolerability profile than retinoids [19,26,27,28,29] that remains to be demonstrated directly.

To define the molecular basis of this protection, skin transcriptomics with gene-set enrichment pointed away from a generic cytosolic antioxidant response and toward mitochondrial oxidative-stress and energy-/oxygen-sensing signatures, identifying the AMPK and HIF-1 pathways as central AKG-responsive cascades alongside suppression of inflammatory programs. This unbiased convergence on mitochondrial and HIF/AMPK-related processes shaped the mechanistic hypothesis tested in vitro and aligns with the view that mitochondrial ROS, rather than generalized oxidative stress, is the dominant pathogenic species in UVB photoaging [8,9,10,11].

Because keratinocytes are the principal epidermal target of UVB and the main site of the identified signatures, we used the HaCaT keratinocyte line to dissect the mechanism under defined conditions, enabling the pharmacological interrogation (DMOG, compound C, MitoTEMPO) that is not feasible in vivo.

In HaCaT cells, AKG attenuated mitochondrial superoxide signals more than total cellular ROS, in contrast to the near-neutral compartmental profile of NAC. This offers a mechanistic explanation for the historically modest efficacy of non-selective antioxidants in photoprotection [41,42] and indicates that engaging endogenous metabolic signaling can approach the compartmental selectivity of an engineered mitochondrial antioxidant (MitoTEMPO [15]) without lipophilic targeting chemistry. The selectivity index reported here is an operational, probe-based comparison rather than a stoichiometric measurement.

A central contribution of this work is the direct demonstration that UVB suppresses, and AKG restores, PHD enzymatic activity—a step previously inferred from HIF-1α protein levels alone [10,24,25]. Because UVB-derived mitochondrial ROS oxidize the active-site Fe(II) [25] while accumulating TCA intermediates such as succinate and fumarate compete with AKG [24,43], PHD inactivation reflects both oxidative inhibition and co-substrate limitation; AKG addresses both, whereas MitoTEMPO relieves only the oxidative component, explaining the greater PHD recovery achieved by AKG. The VHL-dependent prolyl-hydroxylation arm that targets HIF-1α for degradation is well established [22,23,40].

In our model, HIF-1α excess (UVB or DMOG) consistently coincided with low AMPK Thr172 phosphorylation, and HIF-1α clearance by AKG with its restoration, while LKB1 remained constant—indicating that AMPK suppression arises downstream of the constitutive upstream kinase rather than from its loss. We did not, however, demonstrate that HIF-1α directly inhibits AMPK; although HIF-1α stabilization reprograms mitochondrial metabolism in ways that could lower AMPK activity [30,31], a direct effect has not been established and remains to be tested by genetic and phosphoproteomic approaches. We therefore present this step as a proposed, associative link.

These findings also carry implications for mTORC1, a reciprocal node of the energy-sensing network. Activated AMPK restrains mTORC1 through TSC2 and Raptor phosphorylation [16,44] and, independently of AMPK, AKG inhibits TOR signaling [45]; because UVB activates mTOR in skin and mTOR inhibition mitigates UVB damage [46], AKG-driven AMPK reactivation would be predicted to lower mTORC1 activity, consistent with the reduced epidermal hyperplasia observed here. mTORC1 activity was not measured directly and warrants future study.

Impaired degradation is unlikely to be the sole route to HIF-1α accumulation. DEC1 (BHLHE40) is a required upstream mediator of UVB-induced HIF-1α in keratinocytes via an EGFR/PI3K/AKT/DEC1 axis [47]; UVB-induced HIF-1α thus likely reflects both increased DEC1-dependent input and decreased PHD-dependent clearance, with AKG acting predominantly on the latter. Whether AKG also modulates DEC1 warrants future study.

Collectively, AKG is positioned mechanistically apart from both non-selective scavengers (NAC) and engineered mitochondrial antioxidants (MitoTEMPO): rather than neutralizing radicals already formed, it restores the upstream enzymatic and biogenic capacity that limits their production [11,16]. Pharmacological blockade at either α-KGDD (DMOG) or AMPK (compound C) largely abrogated AKG’s protection across mitochondrial superoxide, membrane potential, and ATP, supporting a single upstream architecture rather than parallel independent effects. Relative to ATRA, high-dose AKG achieved comparable structural protection through a distinct, non-RAR mechanism (Figure 8).

Several considerations temper translational interpretation. The microneedle-assisted protocol is itself biologically active, and the thrice-weekly, 10-week regimen does not represent a realistic human-use pattern; cutaneous and intracellular AKG were not directly quantified, and no formal tolerability assessment was performed. With the exception of the mitochondrial functional assays and PHD activity, the AKG-alone arm was not tested across the molecular targets; although AKG alone did not differ from control in those assays, its systematic inclusion for each endpoint would more firmly establish that AKG acts by reversing UVB-induced dysregulation rather than by shifting the basal state. Mechanistically, we relied on pharmacological rather than genetic manipulation (noting that compound C has AMPK-independent effects [48], partially mitigated by our UVB + compound C control); validation in primary and three-dimensional skin models, direct measurement of intracellular AKG, and assessment of other α-KGDD substrates such as TET and Jumonji-domain demethylases [21,49] remain for future work. Mitochondrial biogenesis was inferred from PGC-1α/TFAM together with ΔΨm, ATP, and mtROS rather than from mtDNA copy number or respirometry, and canonical senescence/MMP panels were not assessed. Finally, the chronic 4-MED regimen produced focal superficial erosions in some animals, indicating a combined photoaging/sub-acute-photodamage phenotype that milder regimens may better isolate. Clinical studies in human subjects will be necessary before translation.

## 5. Conclusions

In summary, our study demonstrates that AKG effectively mitigates UVB-induced skin photoaging by orchestrating metabolic and mitochondrial reprogramming. We found that UVB irradiation disrupts the α-KGDD/HIF-1α balance, which appears to function as an upstream regulator of the AMPK-mediated mitochondrial defense system in HaCaT keratinocytes. Our findings suggest that AKG supplementation promotes the degradation of HIF-1α in an α-KGDD-dependent manner, which is associated with restoration of AMPK Thr172 phosphorylation downstream of constitutive LKB1, and concomitant recovery of mitochondrial biogenesis and antioxidant capacity. The precise molecular link between HIF-1α clearance and AMPK reactivation requires further mechanistic dissection. While further clinical validation is required, these results characterize AKG as a potential metabolic intervention to maintain mitochondrial homeostasis and provide a mechanistic basis for its application in preventing UV-induced cutaneous damage.

## Data Availability

The RNA-seq data generated in this study have been deposited in the National Genomic Data Center (NGDC) under accession number PRJCA063446 and will be made publicly available upon publication. All other data supporting the findings of this study are available from the corresponding authors upon reasonable request.

## Supplementary Materials

The following supporting information can be downloaded at https://www.mdpi.com/article/10.3390/antiox15070845/s1. Figure S1: HaCaT keratinocyte UVB dose–response viability and optimization of AKG, DMOG, and compound C working concentrations (CCK-8 assays); Figure S2: secondary non-invasive skin biophysical parameters (elastic recovery time, sebum content, and skin anisotropy) across the six experimental groups; Figure S3: EdU incorporation assay in HaCaT cells (CK, UVB, AKG, UVB + AKG); Figure S4: transcriptomic volcano plots (UVB vs. CK; HAKG vs. UVB) and GSEA enrichment curves.
