# Supplementary material for: Alpha-Ketoglutarate Attenuates UVB-Induced Skin Photoaging by Restoring Mitochondrial Redox Homeostasis
Source: Antioxidants (Basel). 2026 Jul 4;15(7):845. doi: 10.3390/antiox15070845 (PMC13405473; doi:10.3390/antiox15070845)
Supplement: Supplementary file 1 [file antioxidants-15-00845-s001.zip › antioxidants-4334143-supplementary.pdf]

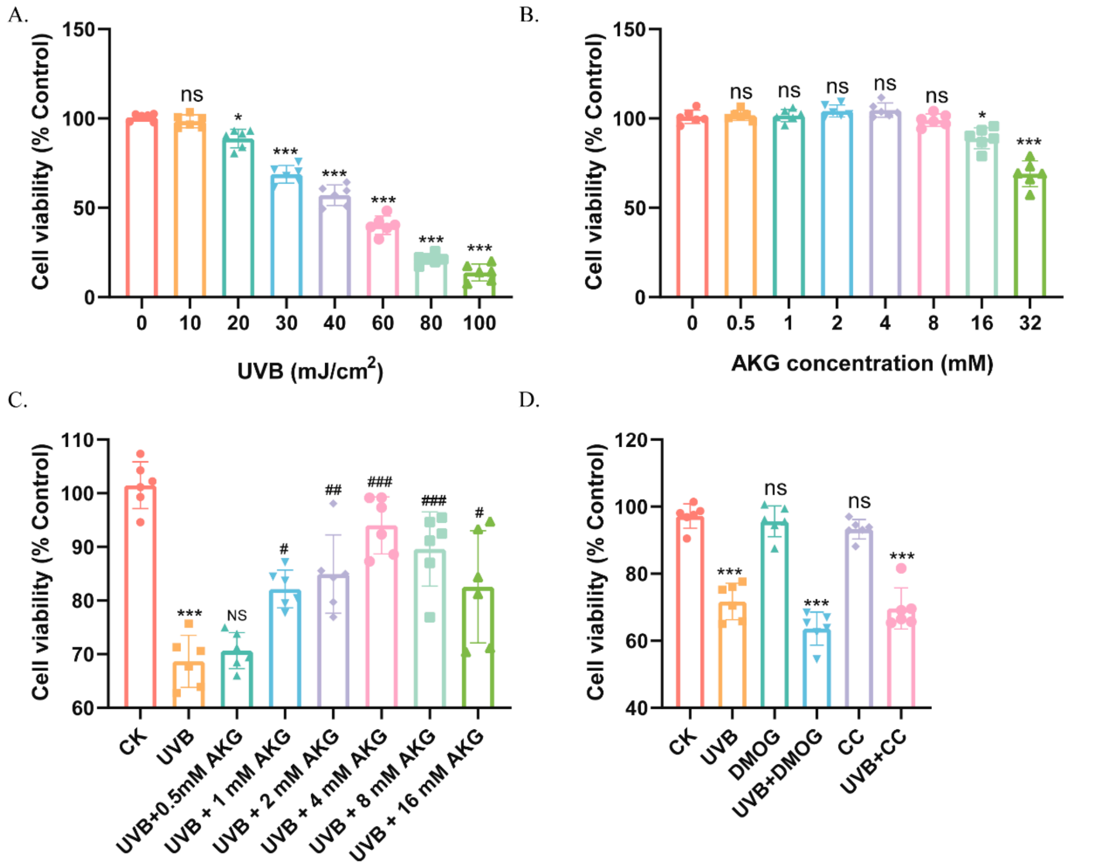

Figure S1. Dose-response characterization of UVB irradiation, α-ketoglutarate (AKG), and pharmacological inhibitors in HaCaT keratinocytes used to define working concentrations for in vitro mechanistic studies. (A) UVB dose-response curve. HaCaT cells were seeded in 96-well plates (8×10^3 cells per well) and exposed the following day to UVB at incremental doses (0, 10, 20, 30, 40, 60, 80, and 100 mJ/cm^2) in PBS, after which the medium was replaced with fresh complete DMEM. Cell viability was determined 24 h post-irradiation by CCK-8 assay and expressed as a percentage of the non-irradiated control. Viability declined in a dose-dependent manner, falling to approximately 70% at 30 mJ/cm^2 (red arrow), to ~55% at 40 mJ/cm^2, and to below 25% at doses ≥80 mJ/cm^2. The dose of 30 mJ/cm^2 was selected for all subsequent in vitro experiments because it elicits sub-lethal stress sufficient to induce oxidative damage and senescence-associated phenotypes while preserving sufficient viable cells for downstream mechanistic analyses, consistent with established HaCaT photoaging protocols [10]. (B) AKG safety profile in non-irradiated cells. HaCaT cells were treated with AKG at the indicated concentrations (0, 0.5, 1, 2, 4, 8, 16, and 32 mM) in pH-adjusted (7.2–7.4) complete medium for 24 h, and viability was assessed by CCK-8 assay. AKG was well tolerated across a broad concentration range, with viability maintained between 99% and 105% of control at concentrations ≤8 mM ($p > 0.05$ vs. untreated control). Mild but statistically significant reductions in viability emerged at 16 mM (~89%) and 32 mM (~71%), likely reflecting osmotic stress associated with sodium counterion accumulation at supraphysiological concentrations. The selected working concentration of 4 mM (red arrow) lies well within the non-cytotoxic range.

(C) Protective effect of AKG against UVB-induced cytotoxicity. HaCaT cells were irradiated with 30 mJ/cm² UVB and immediately treated with AKG at the indicated concentrations (0, 0.5, 1, 2, 4, 8, and 16 mM) in fresh complete medium; viability was assessed 24 h later by CCK-8 assay. AKG dose-dependently rescued cells from UVB-induced viability loss, with the protective effect plateauing between 4 and 8 mM and reaching maximum efficacy at 4 mM (red arrow), which restored viability from approximately 70% (UVB alone) to approximately 92% of non-irradiated control. Higher concentrations (16 mM) showed reduced protective efficacy, consistent with the mild cytotoxicity observed at this concentration in non-irradiated cells (panel B). On the basis of this dose-response profile, 4 mM AKG was selected as the working concentration for all subsequent in vitro experiments. (D) Cytotoxicity assessment of pharmacological inhibitors at the working concentrations used in mechanistic experiments. HaCaT cells were treated with dimethyloxalylglycine (DMOG, 1 mM, an α-KGDD inhibitor) or compound C (CC, 10 μM, an AMPK inhibitor) for 24 h, either alone or in combination with 30 mJ/cm² UVB irradiation, and viability was assessed by CCK-8 assay. Neither inhibitor produced significant cytotoxicity at the working concentrations under non-irradiated conditions (DMOG alone: ~96%; CC alone: ~94%; both $p > 0.05$ vs. vehicle control), confirming the absence of confounding cytotoxic effects independent of UVB-induced damage. Co-treatment of either inhibitor with UVB did not significantly aggravate UVB-induced viability loss compared with UVB alone ($p > 0.05$), indicating that the mechanistic effects of these inhibitors observed in subsequent experiments (Figures 6 and 7) reflect on-target pharmacological action rather than additive cytotoxicity. The final DMSO concentration in all treatment groups did not exceed 0.1% (v/v), and equivalent volumes of DMSO were added to all control groups as vehicle controls. Data are presented as mean ± SD from three independent biological replicates, each performed with six technical replicates per condition (n = 6 wells per condition × 3 independent experiments). Statistical comparisons were performed by one-way ANOVA followed by Tukey's post hoc test. *$p < 0.05$, **$p < 0.01$, ***$p < 0.001$ vs. untreated control; #$p < 0.05$, ##$p < 0.01$, ###$p < 0.001$ vs. UVB-only group; ns, not significant vs. CK; NS, not significant vs. UVB.

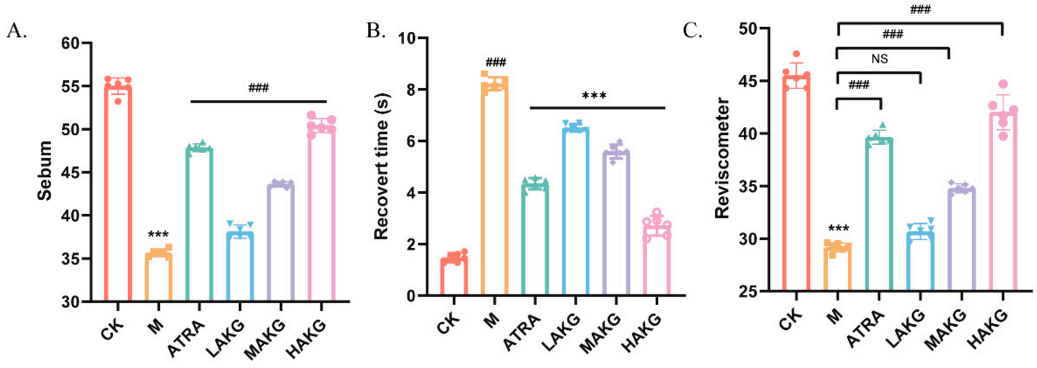

Figure S2. Secondary non-invasive skin biophysical parameters in UVB-irradiated hairless mice at the 10-week endpoint. Complementary to the primary barrier indices shown in Figure 1, three additional skin physiological parameters were measured on

the dorsal skin of anesthetized mice using a multi-probe adapter system (MPA, Courage + Khazaka Electronic GmbH, Cologne, Germany): (A) sebum index (Sebumeter® SM 815), (B) skin elastic recovery time (s; Cutometer® MPA 580), and (C) skin anisotropy (Reviscometer® RVM 600), across the six experimental groups (Control/CK; UVB model/M; ATRA positive control; and low-, medium-, and high-dose AKG [LAKG, MAKG, HAKG]). Each parameter was measured at three randomized sites per mouse and averaged by a single blinded operator. Chronic UVB exposure altered all three indices relative to CK, and topical AKG dose-dependently restored sebum index, elastic recovery time, and skin anisotropy toward control levels, with efficacy comparable to ATRA. Data are presented as mean ± SEM (n = 6). *p < 0.05, **p < 0.01, ***p < 0.001 vs. CK group; #p < 0.05, ##p < 0.01, ###p < 0.001 vs. UVB/M group; ns, not significant.

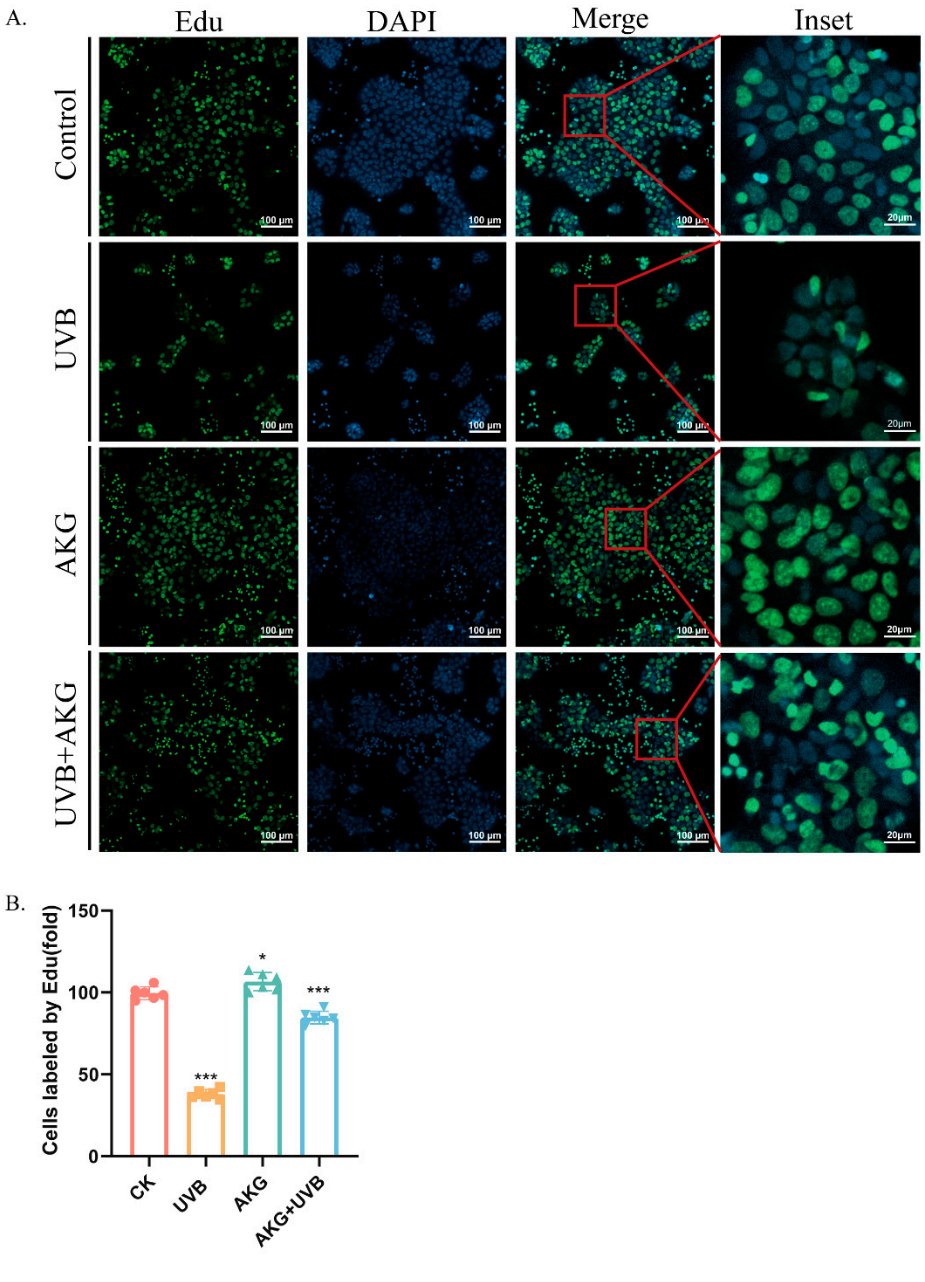

Figure S3. AKG restores S-phase DNA-replicative activity in UVB-exposed HaCaT keratinocytes (EdU incorporation assay). HaCaT cells were assigned to four groups—untreated control (CK), UVB (30 mJ/cm^2), AKG alone (4 mM), and UVB + AKG—and proliferating (S-phase) cells were labeled with 5-ethynyl-2′-deoxyuridine (EdU), detected by click-chemistry fluorescence, and counterstained with Hoechst 33342 to visualize total nuclei. (A) Representative fluorescence images of EdU-positive nuclei (green) merged with Hoechst (blue) for each group (scale bars: main images = 100 μm; insets = 25 μm). (B) Quantification of the EdU-positive fraction (% of total Hoechst^+ nuclei), determined across at least five random fields per condition using ImageJ. UVB markedly reduced the EdU^+ fraction, which co-treatment with AKG significantly restored, whereas AKG alone did not differ from CK, indicating that AKG rescues UVB-suppressed keratinocyte proliferation without perturbing the basal proliferative state. Data are presented as mean ± SD (n = 3 independent experiments). *$p < 0.05$, ***$p < 0.001$ vs. CK group; ns, not significant.

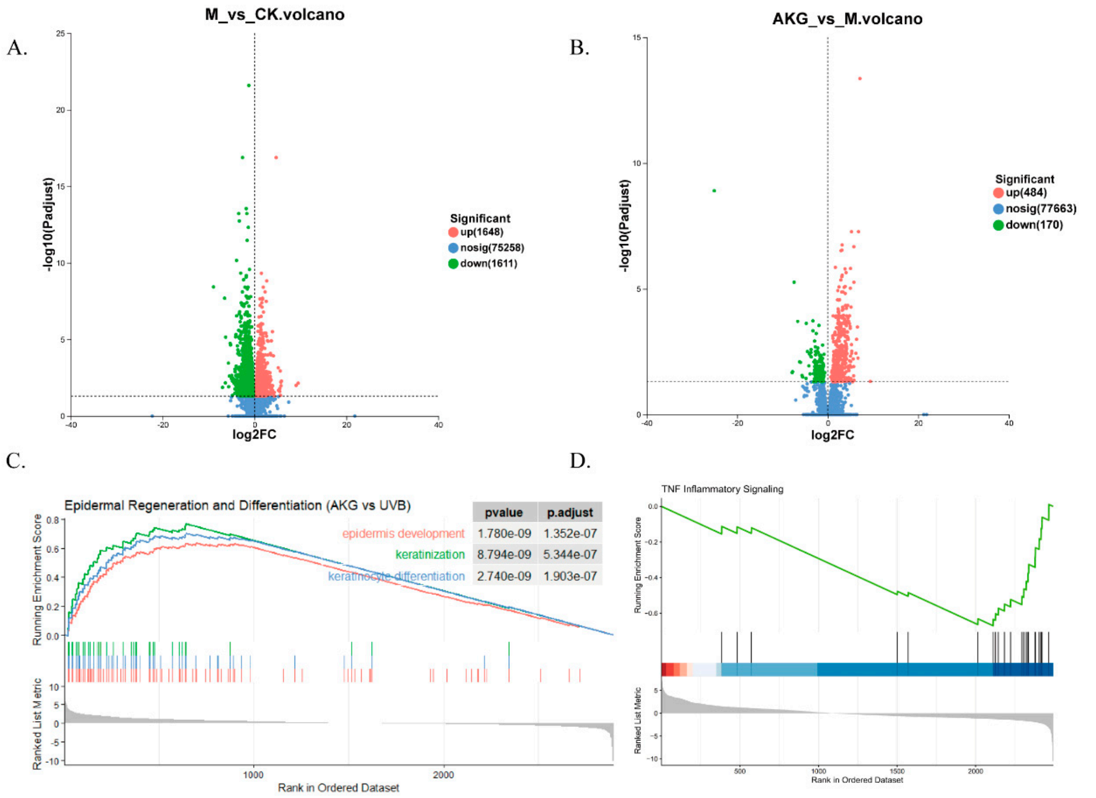

Figure S4. Transcriptomic profiling of UVB-induced and AKG-rescued gene expression in mouse skin (RNA-seq). Total RNA from dorsal skin of the CK, UVB, and high-dose AKG (HAKG) groups (n = 4 per group) was sequenced and analyzed as described in Section 2.9. (A–B) Volcano plots of differentially expressed genes (DEGs; $|\log_2(\text{fold change})| > 1$ and FDR < 0.05, Benjamini–Hochberg): (A) UVB vs. CK and (B) HAKG vs. UVB; up- and down-regulated genes are shown in red and green, respectively. (C–D) Gene-set enrichment analysis (GSEA) enrichment plots for the HAKG vs. UVB comparison: (C) significant positive enrichment of epidermal

regeneration and differentiation gene sets, including "epidermis development," "keratinization," and "keratinocyte differentiation" (all p.adjust $< 1 \times 10^{-6}$); and (D) significant suppression of TNF inflammatory signaling. Normalized enrichment score (NES) and FDR (p.adjust) are indicated for each gene set (significance threshold NES > 1.5, FDR < 0.05).
